# Idiopathic Intracranial Hypertension Presenting With Reversible Hemiparesis: A Rare Stroke Mimic

**DOI:** 10.7759/cureus.102815

**Published:** 2026-02-02

**Authors:** Muhamad Asraf Azhari, Noor Azimah Muhammad

**Affiliations:** 1 Department of Family Medicine, Hospital Canselor Tuanku Muhriz UKM, Kuala Lumpur, MYS

**Keywords:** acetazolamide, cerebrospinal fluid pressure, hemiparesis, idiopathic intracranial hypertension, papilledema, stroke mimic

## Abstract

Idiopathic intracranial hypertension (IIH) presenting with focal neurological deficits is rare and may mimic acute stroke, complicating timely diagnosis. We report the case of a 43-year-old Malay woman with progressive headache, intermittent visual symptoms, and acute-onset, progressive right-sided weakness over two days. Dilated fundus examination revealed bilateral grade 2 papilledema, and neurological examination showed mild hemiparesis. Non-contrast CT and CT angiography of the brain were normal. Lumbar puncture (LP) demonstrated elevated opening pressure with normal CSF findings. She improved significantly following LP and acetazolamide, with complete resolution of hemiparesis within one week. This uncommon presentation highlights the importance of prompt neuroimaging, early ophthalmological assessment, and LP. IIH should be considered in patients with persistent headache and acute hemiparesis once structural and vascular causes have been excluded.

## Introduction

Idiopathic intracranial hypertension (IIH), or pseudotumor cerebri, is a neurological disorder characterized by elevated intracranial pressure (ICP) without identifiable structural, infectious, or vascular causes and with normal CSF composition [1,2]. It predominantly affects obese women of reproductive age, and its prevalence increased approximately sixfold, from 12 to 76 per 100,000, between 2003 and 2017, paralleling rising obesity rates [3]. IIH classically presents with features of raised ICP, such as headache, visual disturbances, and papilledema, which may lead to significant morbidity, including permanent visual impairment or blindness [4]. Focal neurological deficits other than sixth nerve palsy, such as hemiparesis, are an uncommon presentation of IIH and are not well documented in the literature [5-7]. This case, diagnosed as a stroke mimic, adds to the limited reports describing reversible, pressure-mediated hemiparesis in IIH and highlights the importance of thorough evaluation by attending clinicians.

## Case presentation

This case report describes a 43-year-old Malay woman who presented to a clinic with worsening, throbbing bilateral frontotemporal headaches occurring in the early morning over two weeks. The headaches were associated with nausea and recurrent, transient bilateral blurred vision. She also developed progressive right-sided hemiparesis over two days, beginning with reduced right-hand grip strength, followed by leg weakness causing difficulty climbing stairs, and eventually requiring a walking aid for ambulation. She had been on regular medications for hypertension and dyslipidemia for two years and also had obesity, with a BMI of 41 kg/m².

At presentation, she was alert and fully oriented, with normal speech and cognition. She was afebrile, with a blood pressure of 140/80 mmHg and a regular pulse of 70 beats per minute. Cranial nerve examination revealed uncorrected visual acuity of 6/6 bilaterally, intact pupillary light reflexes with no relative afferent pupillary defect, full extraocular movements, preserved facial symmetry and sensation, intact bilateral hearing, and normal tongue and palate movements. Bedside direct fundoscopy was inconclusive, as the optic discs could not be adequately visualized through undilated pupils.

Motor examination demonstrated right-sided hemiparesis with grade 4/5 power, normal muscle tone bilaterally, and no muscle wasting or involuntary movements. Deep tendon reflexes were brisk on the right and normal on the left, with downgoing plantar responses bilaterally. Sensory examination was intact to light touch and pinprick in all limbs. Cerebellar examination revealed no dysmetria or dysdiadochokinesia. Meningeal signs were absent, with no neck stiffness or Kernig’s or Brudzinski’s signs.

**Figure 1:**
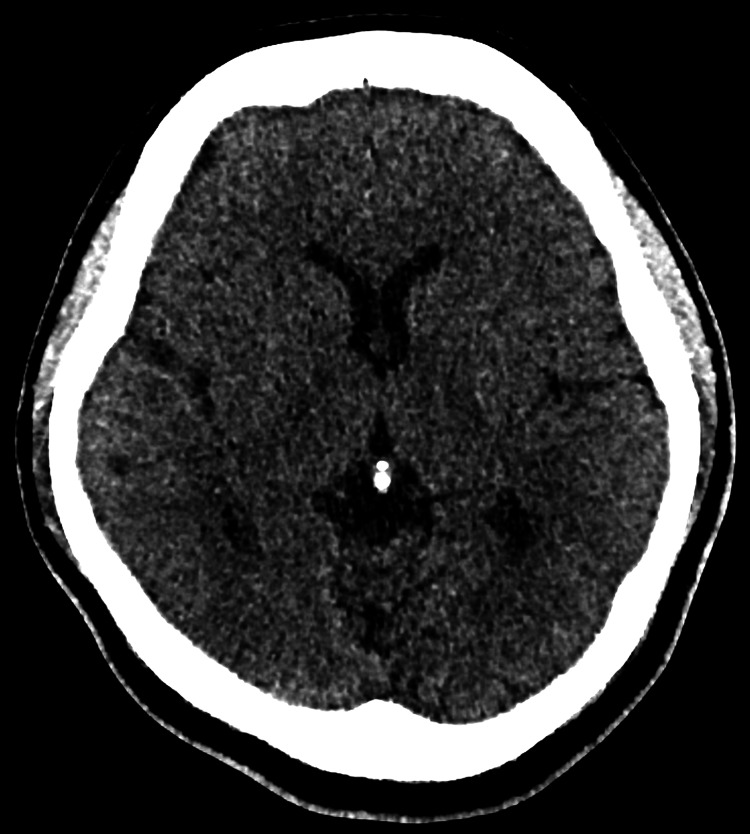
Non-contrast axial brain CT scan demonstrating no acute intracranial abnormalities

**Figure 2:**
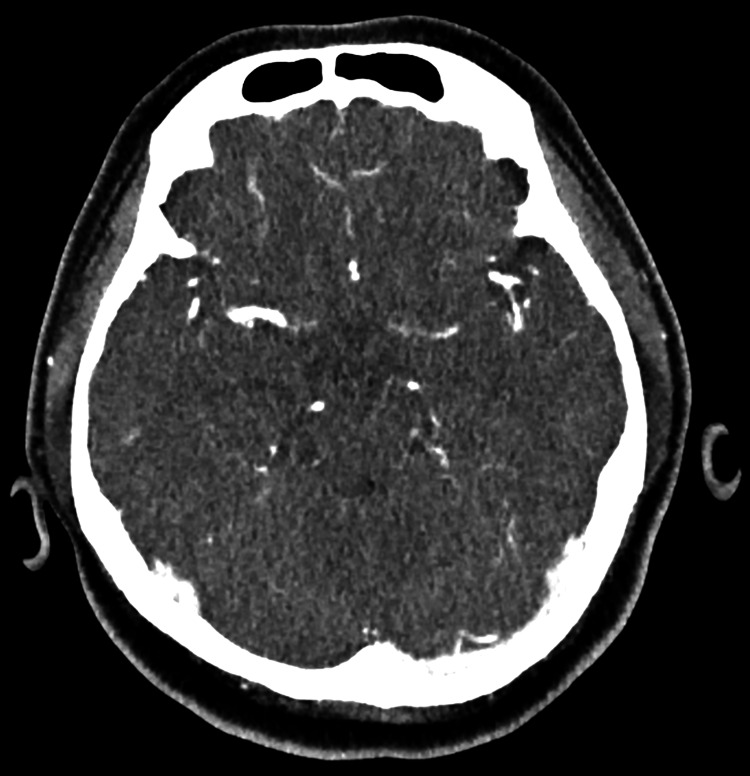
CTA of the brain showing normal arterial opacification with no evidence of large-vessel occlusion

The electrocardiogram showed a normal sinus rhythm without arrhythmia. Initial blood investigations, including random blood glucose, full blood count, renal function, electrolytes, thyroid function, and coagulation profile, were within normal limits. Non-contrast CT of the brain demonstrated no acute intracranial hemorrhage, infarction, mass effect, hydrocephalus, or midline shift (Figure 1). CT angiography (CTA) of the brain showed patent intracranial arteries with no evidence of large-vessel occlusion, stenosis, aneurysmal dilatation, or arteriovenous malformation (Figure 2). The CTA also demonstrated well-opacified dural venous sinuses without filling defects.

**Figure 3:**
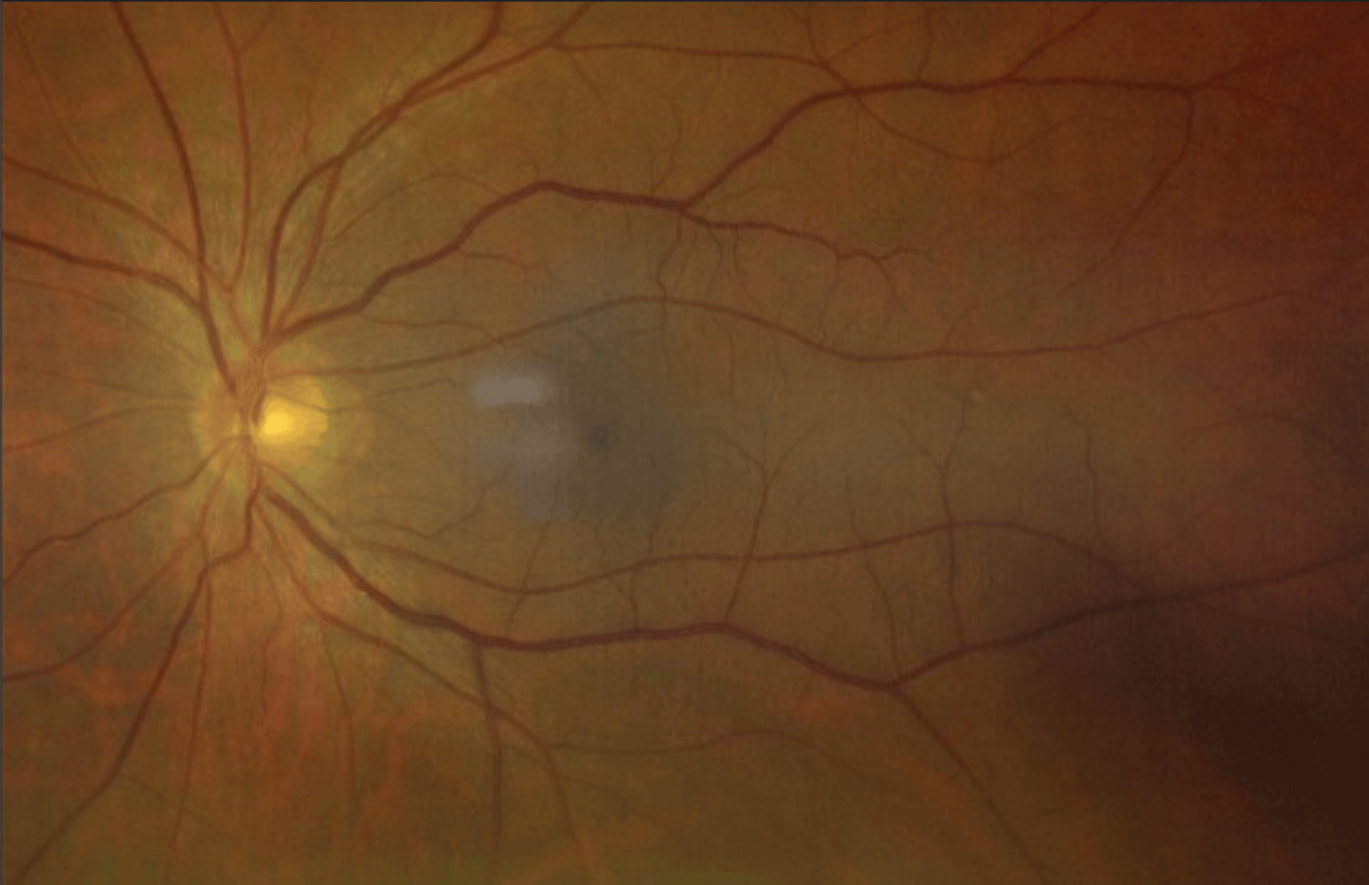
Fundus photograph of the left eye at presentation showing optic disc swelling

**Figure 4:**
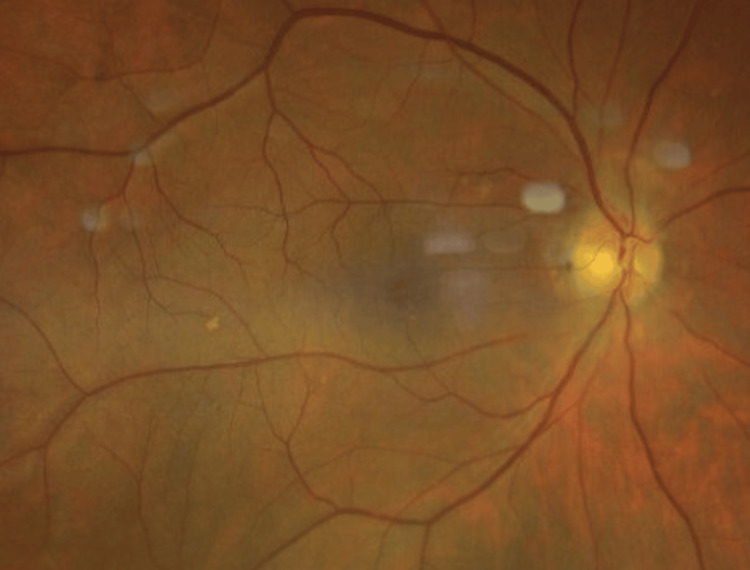
Fundus photograph of the right eye at presentation showing optic disc swelling

Given the history of headache and visual obscurations, an ophthalmology consultation was obtained. Slit-lamp examination revealed no anterior segment abnormalities in either eye. Dilated fundus examination demonstrated bilateral grade 2 papilledema (Frisén scale), with optic disc elevation, circumferential blurring of the disc margins, and a peripapillary halo, without significant involvement of major vessels (Figure 3, Figure 4).

Subsequently, a lumbar puncture (LP) was performed, revealing a markedly elevated opening pressure of 34.5 cm H₂O with normal CSF analysis, including glucose 4.8 mmol/L, protein 319 mg/L, acellular fluid, and no evidence of infection. India ink testing, bacterial antigen testing, acid-fast bacilli staining, and cultures at 48 hours were all negative. CSF cytology was also negative for malignant cells. A total of 18 mL of CSF was drained during the procedure, resulting in immediate relief of the patient’s headache.

She was started on acetazolamide 500 mg twice daily, with progressive resolution of visual obscurations and complete recovery of right-sided hemiparesis within one week of inpatient treatment. The medication was continued upon discharge, with close neurology and ophthalmology follow-up arranged. An outpatient brain MRI and magnetic resonance venography (MRV) were scheduled two weeks post-discharge; however, the patient did not attend the appointment following marked clinical improvement.

At the six-week review, she remained asymptomatic with no recurrence of symptoms, and the acetazolamide dose was reduced to 250 mg twice daily. Given her sustained symptom resolution, stable clinical course, and patient preference following shared decision-making, further neuroimaging was deferred. She remains under close follow-up for monitoring of relapse or complications, with management focused on weight reduction and optimization of vascular risk factors.

## Discussion

This case highlights an unusual presentation of IIH manifesting as hemiparesis. While IIH commonly presents with features of raised ICP, focal motor deficits beyond sixth nerve palsy are rare and should prompt consideration of alternative diagnoses [1]. Only a limited number of case reports have described hemiparesis in IIH, often alongside atypical or migraine-like features, highlighting the rarity of this manifestation [6-8]. In this patient, the combination of normal CT/CTA findings, markedly elevated CSF opening pressure with normal CSF composition, and complete clinical recovery following CSF pressure reduction strongly supports IIH as the diagnosis rather than a cerebrovascular event. Recognition of such an atypical presentation expands the clinical spectrum of IIH and reinforces the importance of considering it among stroke mimics, particularly in obese women.

The pathophysiology of this unusual symptom remains incompletely understood but is thought to result from altered CSF and venous dynamics. In IIH, impaired CSF absorption and reduced venous outflow can lead to sustained ICP elevation [1,5,9]. Such pressure elevation may transiently impair function within motor pathways, plausibly explaining the reversible hemiparesis observed in this patient [5,9]. The complete resolution of motor weakness following LP and acetazolamide supports a pressure-mediated mechanism, consistent with other atypical IIH presentations reported in the literature [6,8].

IIH predominantly affects women of reproductive age and is strongly associated with obesity and recent weight gain [3,5]. Population-based studies have demonstrated that both incidence and prevalence increase in parallel with BMI and weight gain over time [3,5]. Proposed mechanisms include increased intra-abdominal and intrathoracic pressure, venous sinus stenosis, and adipokine-mediated effects on CSF dynamics [5,9]. The patient in this report, an obese middle-aged woman with vascular comorbidities, fits this high-risk profile. Awareness of these risk factors should prompt clinicians to consider IIH in obese women presenting with headache and visual symptoms, even when the initial presentation includes a focal neurological deficit suggestive of stroke [5,6].

At initial presentation, the combination of acute hemiparesis, obesity, hypertension, and dyslipidemia appropriately raised concern for an acute cerebrovascular event. However, when early neuroimaging is unrevealing, alternative diagnoses, including stroke mimics such as IIH, should be considered [5,6]. Features of raised ICP, such as early-morning headache and transient visual obscurations, may be overlooked when attention is focused on focal deficits. Careful fundus assessment is therefore essential, and when bedside fundoscopy is inconclusive, early ophthalmological evaluation is important to detect papilledema and assess visual function [1,5]. Once structural intracranial pathology is excluded, LP remains a cornerstone investigation, with revised criteria emphasizing elevated opening pressure and normal CSF composition in the presence of raised ICP features [2]. In this patient, papilledema on dilated fundus examination, elevated opening pressure, normal CSF profile, and the absence of acute intracranial pathology on CT/CTA supported a diagnosis consistent with IIH [2]. LP may also provide symptomatic benefit by reducing ICP, as observed in this case [1,5].

Early recognition and treatment of IIH are essential to prevent irreversible complications, particularly permanent visual impairment or blindness [1]. Consensus guidelines recommend prompt initiation of acetazolamide to reduce CSF production, alongside weight reduction as the principal disease-modifying strategy [1,5]. In this patient, LP combined with acetazolamide led to rapid improvement in headache, visual symptoms, and hemiparesis, demonstrating the potential for full recovery when IIH is identified and treated early. Long-term follow-up is crucial, as IIH is associated not only with the risk of recurrent ICP elevation and vision loss but also with increased cardiovascular morbidity in affected women [3,10]. Coordinated multidisciplinary care involving primary care, neurology, ophthalmology, and obesity services is therefore essential to support sustained weight loss, optimize vascular risk factors, monitor visual function, and detect early relapse [1,10].

Although brain MRI with MRV is recommended to exclude structural lesions and cerebral venous sinus thrombosis in suspected IIH [1,2], further neuroimaging was deferred in this case because of rapid and sustained clinical improvement following LP and acetazolamide, the stable clinical course on follow-up, and patient preference after shared decision-making. Initial CT and CTA were unremarkable, with no evidence of acute intracranial hemorrhage, infarction, mass lesions, arterial pathology, or reported venous sinus filling defects. A low threshold for additional MRI/MRV was maintained should symptoms recur or new neurological signs emerge, and the benign clinical trajectory to date supports the working diagnosis of IIH.

## Conclusions

This case highlights IIH as an uncommon stroke mimic presenting with reversible hemiparesis, likely related to pressure effects on motor pathways. Awareness of high-risk features, such as female sex and obesity, is essential. Normal initial neuroimaging may contribute to diagnostic delay; therefore, early ophthalmological assessment and LP are crucial. Prompt CSF pressure reduction combined with acetazolamide led to full recovery and may help prevent serious complications, particularly permanent visual impairment or blindness.
